# Silver-Treated Silk Fibroin Scaffolds for Prevention of Critical Wound Infections

**DOI:** 10.3390/biomimetics9110659

**Published:** 2024-10-29

**Authors:** Federica Paladini, Francesca Russo, Annalia Masi, Carmen Lanzillotti, Alessandro Sannino, Mauro Pollini

**Affiliations:** 1 Department of Experimental Medicine, University of Salento, Via Monteroni, 73100 Lecce, Italy; alessandro.sannino@unisalento.it (A.S.); mauro.pollini@unisalento.it (M.P.); 2 Caresilk S.r.l.s., c/o Dhitech, Via Monteroni, 73100 Lecce, Italy; carmen.lanzillotti@caresilk.it; 3 Department of Engineering for Innovation, University of Salento, Via Monteroni, 73100 Lecce, Italy; francesca.russo@unisalento.it (F.R.); annalia.masi@unisalento.it (A.M.)

**Keywords:** antibacterial silver, tissue engineering, wound healing, infections, silk fibroin

## Abstract

The risk of infections in chronic wounds represents a serious issue, particularly in aged people and in patients affected by diseases such as diabetes and obesity. Moreover, the growing resistance demonstrated by many bacterial strains has significantly reduced the therapeutic options for clinicians and has become a great challenge for the researchers in the definition of novel approaches that promote the wound healing process and reduce the healing time. Tissue engineering approaches based on biomaterials and three-dimensional scaffolds have demonstrated huge potential in supporting cell proliferation; among them, Bombyx mori-derived silk fibroin is a very appealing possibility for the development of devices with regenerative properties for wound healing applications. However, due to the high risk of infections in chronic wounds, an antibacterial treatment is also strongly encouraged for preventing bacterial proliferation at the wound site. In this work, to develop a device with regenerative and antibacterial properties, antibacterial silver coatings were deposited onto silk fibroin scaffolds, and the effect of the treatment in terms of chemical–physical and microbiological properties was investigated. The results demonstrated that the silver treatment improved the mechanical properties of the protein scaffold and provided good antibacterial efficacy against representative bacterial strains in wound infection, namely *Escherichia coli* and antibiotic-resistant *Pseudomonas aeruginosa*.

## 1. Introduction

Approximately 8 million people worldwide are affected by wounds, and in both the USA and European countries, 2% of the population is affected by chronic wounds, with a major incidence in aged people and in diabetic and obese patients [1]. Moreover, infections in chronic wounds delay the healing process [1] and represent a serious challenge in wound care, which is further complicated by the presence of multidrug-resistant microorganisms and bacterial biofilms [2]. One of the most concerning pathogens involved in antibiotic resistance and in the microbiological profile of chronic wound infections is *Pseudomonas aeruginosa*, which is characterized by intrinsic mechanisms of resistance to many classes of antibiotics and by the ability to develop resistance via mutations [3,4]. Along with *Escherichia coli*, *Pseudomonas aeruginosa* is typically associated with infections of deeper skin layers [2] and is involved in the later stages of the infectious process, when chronic wounds develop [5].

The wound dressings commonly used in clinical practice, such as gauzes, absorbent cotton, and bandages can provide only a physical protection of the wound site and have limited efficacy in accelerating the wound healing process and in preventing/treating infections [2,6]. An ideal wound dressing material should maintain a moist environment while removing the excess of exudate; it should protect the wound from contaminations and avoid trauma when the dressing is removed and also provide comfort and good thermal and gas exchange [7,8,9].

Therefore, more technologically advanced dressings with the capability to create a protective environment and release active compounds have been developed, and a range of solutions have been proposed based on tissue engineering approaches [2,6]. Three-dimensional scaffolds can provide the wound with a substrate for new tissue formation and can efficiently shield the damaged skin from external contaminations [6]. Among the most suitable biomaterials for tissue engineering are silk proteins, which are known for their biological properties and for their capability to interact with cells through various cellular events, thus promoting faster wound repair and regeneration [10,11,12,13]. In particular, silk fibroin, derived from the cocoons of *Bombyx mori*, has been recognized as effective in accelerating wound healing by mimicking the extra-cellular matrix (ECM) and by stimulating the growth, proliferation, and migration of different cell lines involved in the healing process [7,14]. Combined with silver nanoparticles (AgNPs), which have demonstrated antimicrobial activity against more than 600 microorganisms, including antibiotic-resistant strains, fungi, and viruses [15,16,17,18], silk fibroin can also be considered as a good candidate for the development of antibacterial scaffolds. Metal and metal oxide nanoparticles are the most promising nanomaterials in the biomedical field; among them, silver and Ag_2_O nanoparticles (NPs) have attracted a great number of studies due to their intrinsic antibacterial activity [19]. Although not fully elucidated, many antibacterial mechanisms have been attributed to metal NPs. Some of them involve physical interactions between the nanoparticle and the negatively charged bacterial wall, which cause damage to the membrane and to cytoplasmic components [20,21,22,23]. Moreover, the antibacterial activity of silver NPs has also been attributed to the adhesion and diffusion of ions inside the cell, which determine the inactivation of the respiratory enzymes, cell disruption, the increased concentration of reactive oxygen species (ROS), and then cellular oxidative stress in microbes [20,22,24]. Along with their broad-spectrum efficacy as an antimicrobial agent, an active role has been attributed to silver nanoparticles in promoting wound healing [25,26], thus further suggesting a synergistic action with silk fibroin in different aspects of the wound healing process.

Aiming at the development of a novel device for wound healing applications with simultaneous regenerative and antibacterial properties, in this work the potential of a silver deposition technology for depositing nanostructured silver coatings on the surface of fibroin scaffolds is investigated. The technology, extensively applied on many medical devices and substrates for biomedical application, has never been tested on a fibroin scaffold; thus, an initial study was also required to verify the compatibility of the process with the specific substrate. Then, the effect of the deposited silver nano-coatings was evaluated in terms of the potential impact on the chemical–physical and biological properties of the fibroin scaffold. Very importantly, the effectiveness of the prototype in terms of antibacterial capability was evaluated, in the function of the biodegradation of the material, on the selected bacterial strains, namely *E. coli* and antibiotic-resistant *P. aeruginosa*, as they are the most representative bacterial strains that are responsible for infections of the deeper tissues in chronic wounds.

## 2. Materials and Methods

Silk fibroin scaffolds, kindly provided by Caresilk S.r.l.s. (Lecce, Italy), were produced according to a proprietary process that was specifically defined to obtain a sponge-like device characterized by a porous structure, improved flexibility, and a longer degradation time than the conventional freeze-dried fibroin scaffolds. Chemicals and reagents, such as silver nitrate, methanol, deionized water, and phosphate buffer saline tablets (PBS), were purchased from Sigma Aldrich (St. Louis, MO, USA). Bacteriological agar and tryptic soy broth were acquired form VWR (Leuven, Belgium).

### 2.1. Silver Deposition Treatment

**Figure 1:**
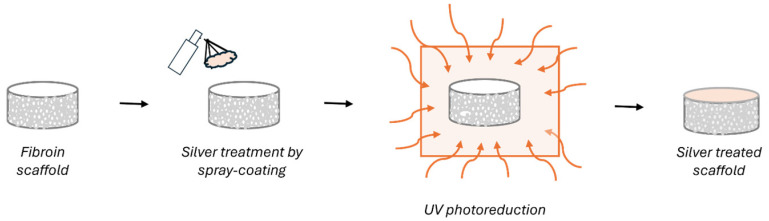
Schematic representation of the silver treatment process performed on fibroin scaffold.

Silk fibroin scaffolds were deposited with silver coatings by using a silver deposition technology based on the in situ photochemical deposition of silver nanoparticles. Briefly, the process consists of (i) the preparation of the silver solution comprising silver nitrate as the nanoparticle precursor, methanol as a photo-reducing agent, and deionized water as a solvent; (ii) the deposition of the silver solution onto the surface of the material through dip coating or spray coating; and (iii) the exposure of the wet substrate to an ultraviolet (UV) source (500 W, λ = 365 nm) in order to allow the conversion from silver salt to metal silver and the simultaneous synthesis of silver nanoparticles directly on the substrate. The composition of the silver solution and, in particular, the percentage of the silver precursor can be properly defined according to the expected antibacterial properties. For the specific application, the silver solution was prepared by mixing 0.1% wt/wt silver nitrate (Sigma Aldrich, ACS reagent, ≥99.0%), 5% wt/wt methanol (Sigma Aldrich ACS reagent, ≥99.8%), and 94.9% wt/wt deionized water at room temperature with magnetic stirring until complete dissolution of the silver salt. The solution was deposited by spray coating on both the top and bottom layers of the scaffolds and then exposed to a UV lamp (Jelosil) for 10 min per side at a distance of 15 cm. After the UV treatment, the samples were placed under the hood overnight for complete drying. A schematic representation of the silver deposition treatment on the silk fibroin scaffolds is shown in Figure 1.

### 2.2. Morphological Characterization

Scanning electron microscopy SEM (Zeiss, EVO, Jena, Germany) was adopted to investigate the initial structure of the scaffolds in terms of pore size and distribution and the potential effect of the silver treatment on these parameters. The morphology was compared between the untreated and silver-treated samples by considering the skin–core distance, which is related to the depth of penetration of the silver treatment.

### 2.3. Mechanical Properties

The mechanical properties of the fibroin scaffolds were evaluated by tensile tests and suture strength tests using a universal testing machine (Zwick Roell, Ulm, Germany) equipped with a 100 N load cell. The samples for the mechanical tests (10 mm × 5 mm) were immersed in phosphate-buffered saline (PBS, 1×) at room temperature for 1 h. For the tensile tests, each sample was locked at 2 mm at both ends. The tests were performed at a displacement speed of 0.1 mm/s and with a load of 0.1 N. The thickness and width of the hydrated samples were measured using a Dino-Lite digital microscope (AnMo Electronics Corporation, New Taipei City, Taiwan). The tensile elongation (ε) and tensile strength (σ) were evaluated in triplicate at the break, and the results were expressed as the mean value ± standard deviation (SD).

The suture strength test was performed to determine the tear strength of the fibroin scaffolds and to simulate a surgical procedure. A single suture was performed at a distance of 2 mm from the top edge. The test was performed by using a glycolic acid copolymer and lactic acid-based suture (Polyglactin 910, VICRYL V311, Johnson & Johnson International, New Brunswick, NJ, USA) using an SH-1 needle and a 3–0 gauge. The wire was fixed to the upper grip of the mechanical test frame; the hydrated samples were tested in triplicate with a load cell of 100 N and an extension speed of 1 mm/min. The maximum load was recorded in Newtons (N) and normalized with respect to the thickness of the scaffold. The results were expressed as a mean value ± SD.

### 2.4. Swelling Tests and Contact Angle

The ability of the scaffolds to absorb liquids was studied by calculating the degree of swelling. The dried scaffolds were cut (10.0 × 5.0 mm^2^), and the dry weight of the scaffolds was recorded before immersion in PBS at 37 °C. The weight of the swollen scaffolds, after the removal of excess water with filter paper, was measured at different time points, up to 24 h. The swelling ratio was calculated using the following equation:

$$SD\;\left(\%\right)=\frac{Ww-Wd}{Wd}\times 100$$

where *Wd* is the weight of the dry scaffolds and *Ww* is the weight of the wet scaffold. The test was performed in triplicate, and the results were expressed as mean value ± SD.

The wettability of the scaffold surface was assessed by measuring the static contact angle using the sessile drop method with a FTA1000 (First Ten Angstroms, Newark, NJ, USA), dropping 10 µL of milli-Q water on 1 cm × 1 cm samples at 25 °C. Four readings were performed for each sample.

### 2.5. Antibacterial Effect

In relation to the potential applications of the device, which is intended to be used in a physiological environment at body temperature and in contact with biological fluids for 3–7 days, the untreated and silver-treated fibroin samples were tested before and after degradation assays. The samples (size 3 × 1 cm^2^, average weight 150 mg) were incubated in 20 mL of PBS at 37 °C and, at the defined time points (0, 1, 3, and 7 days), the samples were characterized in terms of residual antibacterial effect.

The antibacterial capability of the silver-treated samples was determined through both qualitative and quantitative assays after the silver treatment (*t* = 0) and during the degradation experiments at the time points *t* = 1 day, *t* = 3 days, and *t* = 7 days. The selected bacterial strains were *Escherichia coli* (ATCC 25922, inoculating bacterial density 8.0 × 10^6^ CFU/mL) and antibiotic-resistant *Pseudomonas aeruginosa* (ATCC BAA-3285, inoculating bacterial density 2.04 × 10^6^ CFU/mL).

The qualitative assay consists of agar diffusion tests and was performed according to the Standard SNV 195920-1992, which defines the experimental procedure and provides the indications for associating a specific level of antibacterial efficacy to each sample. The experimental and control samples were placed in contact with 200 µL of bacterial suspension onto an agar plate for 24 h at 37 °C (Incubator IGS100, Thermo Scientific Heratherm, Waltham, MA, USA). After incubation, the dimension of the inhibition area to the bacterial growth around and beneath each sample was evaluated and compared with the levels of antibacterial capability provided by the standard. An inhibition area larger than 1 mm indicates an antibacterial efficacy labelled as “good”; a sample completely colonized by bacteria indicates an “insufficient” antibacterial efficacy; intermediate levels, such as “fairly good” or “sufficient”, can be also observed [27,28,29,30].

The quantitative tests were performed through spectrophotometric analyses and optical density (OD) measurement at 600 nm by using a Spectrophotometer (V-1200, VWR, Radnor, PA, USA). In duplicate, the samples (untreated and silver-treated, before and after degradation) were immersed in the bacterial suspension (medium inoculated with each bacterial strain at initial OD = 0.0005) and kept at 37 °C for 4 h. Then, the OD600 was measured and converted to CFU/ml (1 OD = 8 × 10^8^ CFU/mL in the case of *E. coli* and 1 OD = 2.04 × 10^8^ CFU/mL in the case of *P. aeruginosa*). The percentage of antibacterial efficacy (ABE) was calculated in comparison with the control sample according to the following equation [31,32]:

$$ABE\;\left(\%\right)=\frac{Vc-Vt}{Vc}\times 100$$

where *Vc* and *Vt* are the numbers of bacterial colonies grown in the presence of the untreated and treated samples, respectively [33].

### 2.6. Quantification of Fibroin Release Through Bicinchoninic Acid Assay

The release of fibroin in solution was also evaluated through a static degradation experiment and bicinchoninic acid (BCA, Sigma Aldrich) assay after incubation of the samples in phosphate-buffered saline solution at 37 °C for 1, 3, and 7 days. The fibroin protein reduces alkaline Cu (II) to Cu (I) in a concentration-dependent manner. As the bicinchoninic acid is a specific chromogenic reagent for Cu (I), forming a complex with maximum absorbance at 562 nm, the absorbance measured at 562 nm is directly proportional to the fibroin concentration. Bovine serum albumin (BSA) was used as standard protein; the experiments were performed in triplicate, and the results were expressed as the percentage of the residual fibroin with respect to the 100% fibroin scaffold at *t* = 0.

### 2.7. Statistical Analysis

Statistical analysis was performed using the two-tailed Student’s *t* test. Differences were considered significant at *p* < 0.05.

## 3. Results

### 3.1. Silver Deposition Treatment

**Figure 2:**
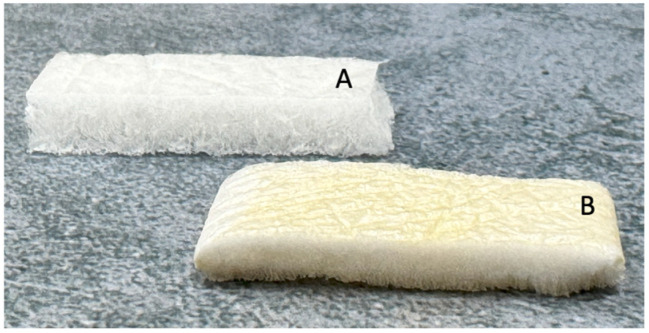
Untreated (**A**) and silver-treated (**B**) fibroin scaffolds.

The prototype of the antibacterial fibroin scaffolds developed in this work (Figure 2) was obtained by treating only the external surfaces of the device as protective layers for preventing bacterial adhesion to the damaged skin. As visible in Figure 2, the treated surfaces are characterized by the typical light browning due to the presence of the silver coating.

The coating resulted in being homogeneous according to a visual inspection, thus also confirming that the spray coating technique was effective for providing a good distribution of the silver solution on this specific substrate. A slight penetration of the silver solution immediately beneath the external layer can also be observed, indicating that a low amount of solution penetrated into the underlaying layer.

### 3.2. Morphological Characterization

**Figure 3:**
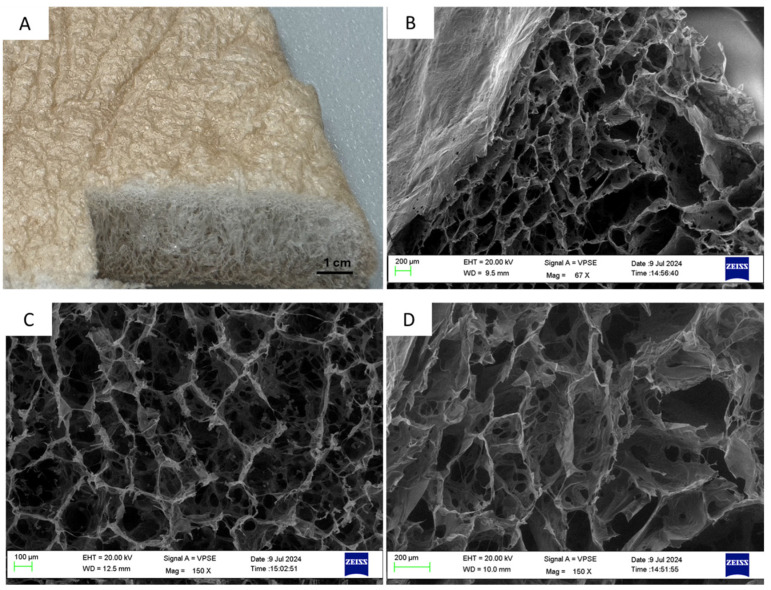
Picture of the silver-treated sample (**A**); SEM image of the silver-treated sample showing the presence of a skin layer and a slightly different porosity between the skin and the core (**B**); similar inner porosity between untreated (**C**) and silver-treated samples (**D**).

The scanning electron microscopy (SEM) analysis is reported in Figure 3A–D, where the skin and the core of the scaffold can be clearly observed. SEM analysis was also performed to investigate the potential effect of the silver treatment on the architecture of the material, and interestingly, a different result was observed between different areas of the scaffold in terms of the function of the distance from the treated layers. While a slight change in pore size and shape was observed in the treated sample in the proximity of the skin (Figure 3B), the porous structure of the scaffold appeared to be unaffected by the silver treatment at the central areas of the samples. The mean pore sizes of the untreated and treated samples (Figure 3C,D), calculated through Image J (ImageJ software 1.53e), resulted in being 331.28 μm and 302.42 μm, respectively. This indicated that the silver solution or the UV photo-reduction may have influenced the porosity, with an effect limited to the outer regions of the scaffold.

### 3.3. Mechanical Properties

**Figure 4:**
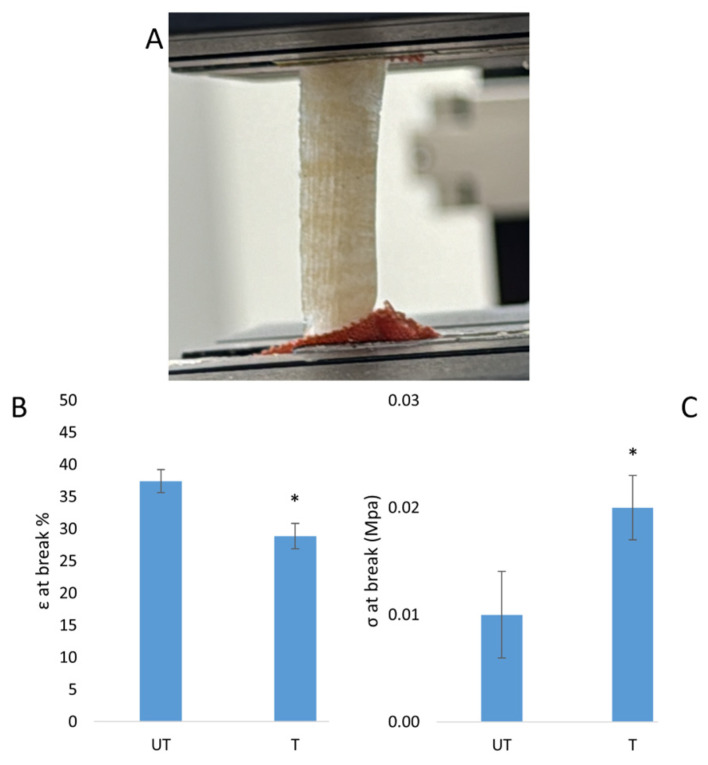
Tensile tests performed on untreated (UT) and silver-treated (T) scaffolds. Image of the sample during the test (**A**); graph reporting the results of the tensile test as elongation at break (**B**) and σ at break (**C**) (*t*-test, *n* = 3, * = *p* < 0.05).

**Figure 5:**
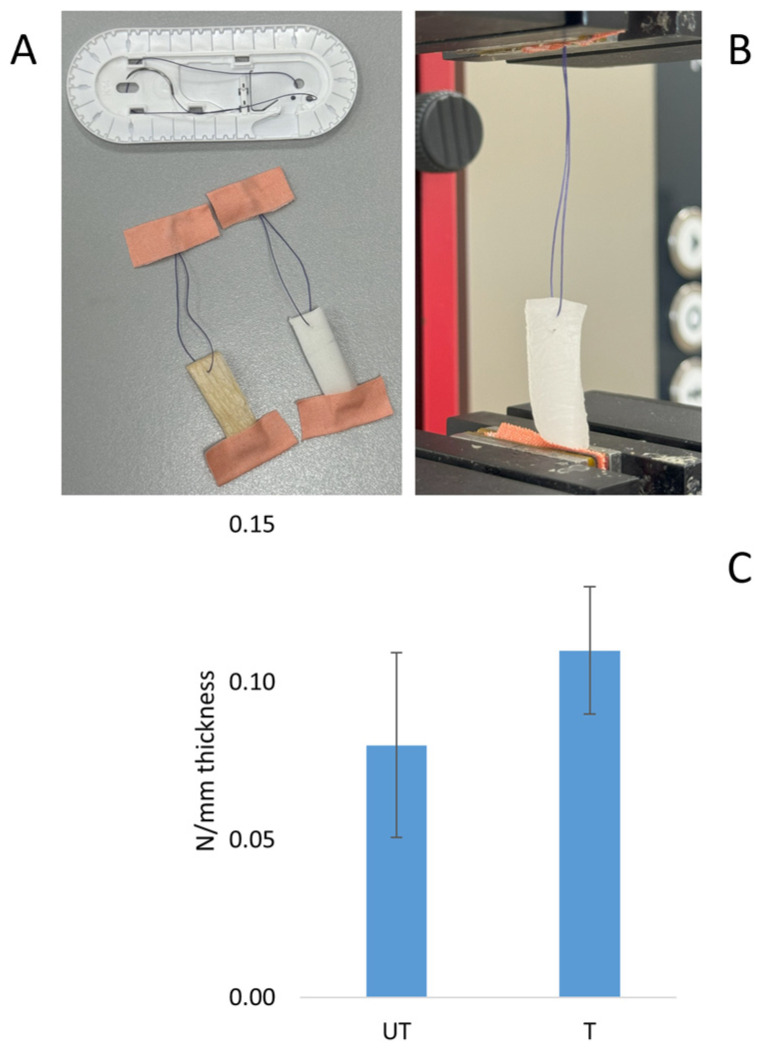
Tensile tests performed using sutures: preparation of samples with suture thread (**A**); sample inserted into the machine grippers (**B**); graph reporting breaking strength (**C**) (*t*-test, *n* = 3, *p* > 0.05).

An effect of the silver treatment on the mechanical properties of the fibroin scaffold was observed in comparison with the untreated material, in terms of both elongation and tensile strength at the break. The average epsilon (ε) was 37.41% for the untreated sample and 28.86% for the treated sample, while the tensile strength was 0.01 MPa for the untreated sample and 0.02 MPa for the silver-treated sample (Figure 4). The values obtained by the suture tests were 0.08 N/mm and 0.11 N/mm for the untreated sample and the silver-treated sample, respectively (Figure 5).

### 3.4. Swelling Tests and Contact Angle

**Figure 6:**
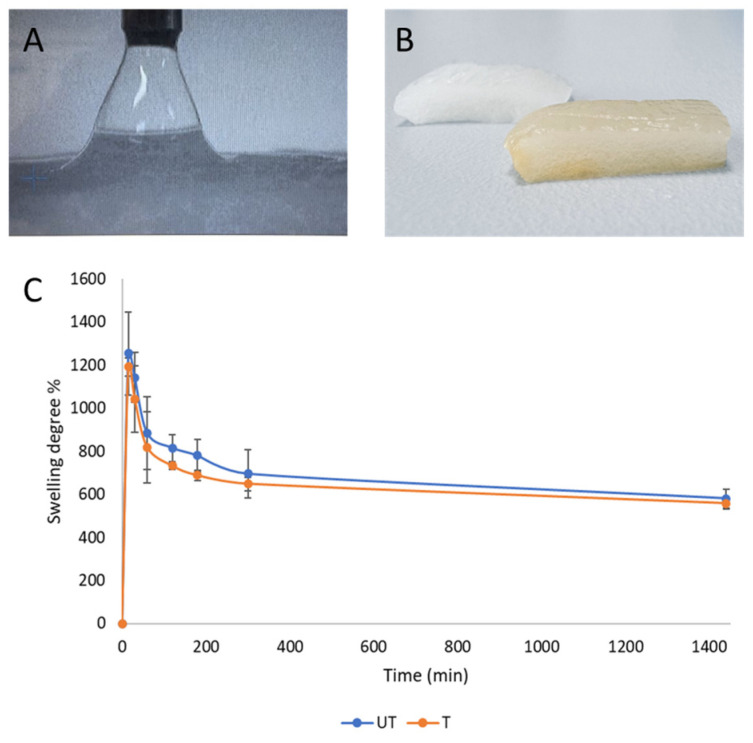
Swelling test: image of the droplet immediately absorbed by the sample (**A**); picture of the samples after the swelling test (**B**); percentage of swelling degree (**C**) calculated for the untreated (UT) and silver-treated (T) samples (*t*-test, *n* = 3, *p* > 0.05).

The hydrophilicity of the material was still very high, as confirmed by the contact angle measurements and also by the complete and fast absorption of the water droplet in the presence of the silver coating (inset of Figure 6). The swelling tests demonstrated the high absorption capability of both the samples, with a slight difference between the untreated and treated samples (Figure 6), which can still be associated with the partial crystallization of the protein. As expected, the highest values of the swelling degree corresponded to the initial time points and decreased during the experiment, still maintaining the absorption properties even after 24 h. The high absorption properties represent a great advantage in terms of the management of exudate in critical wounds and the prevention of exudate contamination by bacteria.

### 3.5. Antibacterial Effect

The residual antibacterial capability was evaluated by simulating the application of the device in a biological environment at high risk of infection.

**Figure 7:**
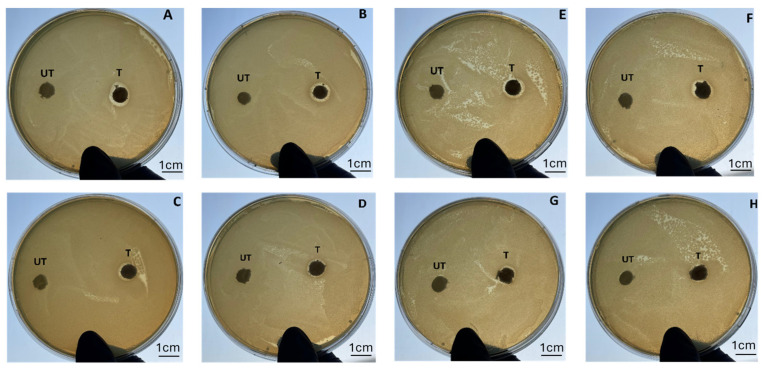
Agar diffusion tests performed on silver-treated fibroin scaffolds (T) and control samples (UT) with *E. coli* (**A**–**D**) and antibiotic-resistant *P. aeruginosa* (**E**–**H**) at different degradation time points, namely *t* = 0 (**A**,**E**), *t* = 1 day (**B**,**F**), *t* = 3 days (**C**,**G**), and 7 days (**D**,**H**).

The microbiology characterizations were performed through both qualitative and quantitative assays of two strains of bacteria, namely *E. coli* as a representative microorganism associated with multiple types of infections, and *P. aeruginosa*, which is one of the most common bacterial pathogens in chronic wounds and is known for its propensity to form biofilms and evade conventional treatment methods [34]; thus, it was selected in this work in an antibiotic-resistant strain. The results of the agar diffusion test (Figure 7) indicated a good antibacterial capability of the silver-treated samples against both of the bacterial strains, according to the levels of antibacterial activity provided by the standard [27,28,29,30]. As expected, the highest antibacterial effectiveness could be observed before the degradation test at *t* = 0, and interestingly, it was still maintained over the entire degradation experiment, with an inhibition area larger than 1 mm (Figure 7).

The quantitative tests confirmed these results and allowed the quantification of the antibacterial efficacy in terms of the reduction in bacterial proliferation. Compared with the sample at *t* = 0, which demonstrated 95% reduction for *E. coli* and 92% for antibiotic-resistant *P. aeruginosa*, the following time points confirmed that, despite the very low amount of silver, the treatment was effective in providing a long-term antibacterial capability. The values are reported in Table 1, where, interestingly, a similar efficacy can be also observed for both of the bacterial strains.

### 3.6. Quantification of Fibroin Release Through Bicinchoninic Acid Assay

**Figure 8:**
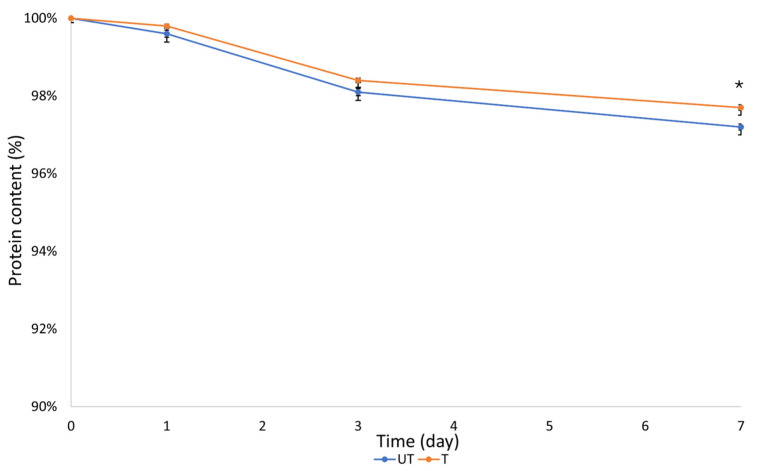
Residual percentage of protein content obtained through BCA at different time points (* = *p* < 0.05 at 7 days).

At the same time points, the loss of fibroin from the scaffolds was quantified through the BCA assay. Figure 8 reports the residual fibroin content from the scaffold up to one week of degradation, with a loss of protein estimated at about 2.8% wt in the untreated sample and 2.3% wt in the silver-treated sample.

This confirmed that the scaffold can be considered stable until the potential replacement of the device and that the silver treatment provides, along with the antibacterial properties, improved performances.

## 4. Discussion

Aiming at the development of a novel device with simultaneous regenerative and antibacterial properties for the treatment of critical wounds, in this work silk fibroin scaffolds were treated using a silver deposition technology, which provided the in situ synthesis and deposition of Ag nanoparticles characterized by a strong adhesion to the substrate. Indeed, previous works have demonstrated that this technology is effective in providing a uniform nanostructured silver coating on multiple types of natural and synthetic substrates, such as medical devices, including surgical sutures and catheters, and textile materials, including cotton gauzes and medical flax [35,36]. The long-term antimicrobial capability of these materials has been assessed on Gram-positive and Gram-negative bacteria, Methicillin-resistant *Staphylococcus aureus* (MRSA), and fungi, even after the aging of the samples [37]. However, the technology had never been tested on fibroin scaffolds; thus, a preliminary study was required to determine the proper process parameters to avoid a significant alteration in the structure and properties of the protein-based device. In particular, the scaffolds kindly provided by Caresilk S.r.l.s. for this research were specifically designed to provide a wound dressing biomaterial with improved stability in terms of biodegradation time, which represents a great advantage for clinical applications in terms of the replacement of the device at the wound site and prolonged bioactivity. Fibroin protein at the wound site can represent a boost for wound healing due to its well-known tissue regenerative properties. At the same time, the three-dimensional porous structure represents the ideal substrate for cell growth and proliferation. On the other hand, critical wounds can be colonized by microorganisms and become infected, thus delaying the wound healing process. The prevention of infection represents an important issue [38,39] that significantly contributes to wound healing, and among the natural antimicrobials, silver has been incorporated in many products in different forms. Some interesting articles in the literature reported the development of hybrid systems based on biodegradable materials/silver nanoparticles for antibacterial application, with a focus on the effect of the incorporation of silver on the properties of polymers. For example, Fortunati et al. developed nanocomposite films based on poly (DL-Lactide-co-Glycolide) copolymer (PLGA) and different concentrations of silver nanoparticles by solvent casting. The results obtained by the authors demonstrated the potential of biodegradable polymers combined with silver nanoparticles in terms of tunable properties and antimicrobial capability for biomedical applications. The degradation mechanism of PLGA in the nanocomposite was not affected by Ag NPs, which also provided a stabilizing effect in the material [40]. In poly(vinyl alcohol (PVA), the addition of fibroin and silver nanoparticles influenced the stiffness of the PVA membranes obtained by electrospinning, improved the thermal stability, and decreased the contact angle due to the hydrophilic nature of fibroin [41]. The hybrid composite based on electrospun silk fibroin/cellulose acetate gold/silver NPS developed by Arumugan et al., where silk fibroin and cellulose acetate represented a stabilizing agent for metal ions, demonstrated excellent biological activity [42]. Wang et al. developed a multifunctional antibacterial composite film based on cellulose acetate/fibroin, which was selected as a biodegradable eco-friendly substrate for providing sites for the in situ deposition of Ag@AgCl nanoparticles. The functional groups in cellulose acetate and silk fibroin, along with the porous structure of the composite film, provided tight immobilization of the silver ions and decreased their accumulation [43]. Based on the consideration of a potential effect of the silver treatment on the properties of the fibroin scaffold, this research aimed to investigate the influence of the adopted silver deposition technology on the features of the fibroin-based device in terms of morphology, mechanical properties, antibacterial activity, swelling, and protein release. In particular, aiming at developing a device with simultaneous antibacterial and regenerative properties for supporting the dynamic wound healing process in both the inflammatory and proliferative phases, the device was tested at the time points of 0, 1, 3, and 7 days, assuming a change in the wound dressing after one week from the injury. Interestingly, the silver treatment was demonstrated to have an effect on some properties of the fibroin scaffold, which can be mainly attributed to the presence of methanol in the silver solution. Although a very low percentage was used, methanol may have partially induced a crystallization of fibroin at the skin sides, which, in the silver-treated samples, appeared stiffer than in the untreated ones. Indeed, methanol or potassium chloride has been reported to be responsible for conversion from silk I to silk II, which represent, respectively, a crystalline metastable structure (silk I), including bound water molecules, and a most stable structure (silk II), with higher mechanical properties due to hydrogen bonding between the peptide blocks [12]. Although some properties did not change in the treated samples, such as the inner porosity (Figure 2), the swelling properties, and the hydrophilicity (Figure 6), other features were improved by the presence of the silver coating. In particular, lower elongation at the break and higher tensile strength were observed during the tensile tests, while the results of the suture tests were approximately the same. Moreover, the potentially increased crystallization attributed to the methanol might also have influenced the biodegradation of fibroin, which remained almost unaltered until one week; at 7 days, it revealed a major stability in the silver-treated samples. This can be considered advantageous in terms of the need for the replacement of the device and for regenerative and antibacterial application. Indeed, the microbiological characterization confirmed the efficacy of the device up to one week, through both the qualitative and quantitative tests performed using relevant bacterial strains for wound infection, such as *E. coli* and antibiotic-resistant *P. aeruginosa*. The results demonstrated that the developed device could be considered a good option for clinical practice.

## 5. Conclusions

In this work, a novel silver/fibroin-based device was developed for wound healing applications. In particular, a fibroin scaffold characterized by improved stability was selected as a substrate for the deposition of a silver coating onto the external surfaces, in order to combine the biological properties of the silk protein with the antimicrobial properties of silver. The characterizations of the treated materials were addressed in the evaluation of a potential effect of the silver treatment on the properties of the fibroin scaffolds, and they demonstrated improved mechanical properties and biodegradation without influencing the inner porosity and the hydrophilicity. The microbiological tests confirmed the good antibacterial efficacy of the treated devices, even after one week and against an antibiotic-resistant bacterial strain, thus suggesting that the developed device could be considered a good option for supporting the wound healing process.

## Figures and Tables

**Table 1:** Antibacterial efficacy (ABE)% calculated through quantitative tests.

Bacteria	Degradation Days	Materials	Initial CFU	CFU After 3 h	ABE (%)
*E. coli*	0 days	Untreated Ag/silk scaffold	4 × 10^5^ 4 × 10^5^	3.0 × 10^8^ 1.6 × 10^7^	- 95
	1 days	Untreated Ag/silk scaffold	4 × 10^5^ 4 × 10^5^	2.7 × 10^8^ 1.8 × 10^7^	- 93
	3 days	Untreated Ag/silk scaffold	4 × 10^5^ 4 × 10^5^	2.6 × 10^8^ 2.0 × 10^7^	- 92
	7 days	Untreated Ag/silk scaffold	4 × 10^5^ 4 × 10^5^	2.8 × 10^8^ 3.2 × 10^7^	- 89
*P. aeruginosa*	0 days	Untreated Ag/silk scaffold	1.02 × 10^5^ 1.02 × 10^5^	4.1 × 10^7^ 3.4 × 10^6^	- 92
	1 days	Untreated Ag/silk scaffold	1.02 × 10^5^ 1.02 × 10^5^	4.2 × 10^7^ 4.1 × 10^6^	- 90
	3 days	Untreated Ag/silk scaffold	1.02 × 10^5^ 1.02 × 10^5^	3.7 × 10^7^ 4.6 × 10^6^	- 88
	7 days	Untreated Ag/silk scaffold	1.02 × 10^5^ 1.02 × 10^5^	4.7 × 10^7^ 6.1 × 10^6^	- 87

## Data Availability

Data are contained within the article.
